# Sex-associated transcriptional changes to synovial macrophages in the aging joint

**DOI:** 10.3389/fimmu.2026.1724385

**Published:** 2026-03-03

**Authors:** Matthew Dapas, Erica N. DeJong, Yidan Wang, Cally Mills, Samuel D. Dowling, Meghan L. Mayer, Tyler Therron, Samuel D. Hamilton, Carla M. Cuda, Dawn M. E. Bowdish, Deborah R. Winter

**Affiliations:** 1 Division of Rheumatology, Department of Pediatrics, Northwestern University Feinberg School of Medicine, Chicago, IL, United States; 2 Center for Human Immunobiology, Feinberg School of Medicine, Chicago, IL, United States; 3 Department of Medicine, McMaster University, Hamilton, ON, Canada; 4 Feinberg School of Medicine, Department of Pediatrics, Division of Rheumatology, Northwestern University, Chicago, IL, United States

**Keywords:** aging, CITE-seq, joints, macrophage, synovial macrophages

## Abstract

**Background:**

Synovial macrophages are critical to tissue maintenance and immune homeostasis in the joints. However, the function of synovial macrophages is compromised with age, leading to increased susceptibility to chronic inflammation and arthritis. Here, we compare the transcriptional heterogeneity of synovial macrophages in young and old joints from male and female mice to better understand the impact of aging and the role of sex.

**Methods:**

We compared synovial macrophage composition and transcriptional profiles in young vs. old joints from male and female mice using single-cell RNA sequencing with cell-surface protein detection (CITE-seq).

**Results:**

We defined five major synovial macrophage subpopulations: CX3CR1+ lining, CD163+ interstitial, MHCII+ monocyte-derived, Ly6C+ infiltrating, and *Ctsk*-expressing osteoclast-like cells, across age and sex. Ly6C+ macrophages were expanded in old mice of both sexes compared to young, while CX3CR1+ lining macrophages were reduced. MHCII+ macrophage proportion differed between sexes, with *Arg1*-expressing cells driving an increase in females and a decrease in males with age. Age-associated differential expression was positively correlated between sexes in the CX3CR1+ and CD163+ subpopulations and negatively correlated in the MHCII+ subpopulations. Significantly enriched pathways included upregulated electron transport chain signaling in interstitial macrophages in both sexes and significantly downregulated MAPK signaling in *Ctsk*+ macrophages in females. Trajectory analysis suggested that the aging synovial macrophage compartment was depleted for transitional cells, which may indicate a macrophage differentiation defect.

**Conclusions:**

In summary, we report on both conserved age-related changes and those that differed between males and females. Our results provide insights on how macrophage heterogeneity changes with age in a sex-dimorphic manner and lays the foundation for research into their role in age-associated diseases, such as arthritis.

## Introduction

Macrophages reside in the tissue of all synovial joints in the body, including ankles and knees. Synovial macrophages are critical to joint health through maintaining tissue homeostasis and structural integrity. On the other hand, synovial macrophages can drive the development of joint inflammation associated with arthritis (1). Macrophages also contribute to cartilage degradation (2, 3) and are drivers in mouse models of both osteoarthritis (OA) (4–7) and rheumatoid arthritis (RA) (8–12). Similarly, studies using clinical samples have implicated synovial macrophages as potential sources of diagnostic and response biomarkers in OA and RA (13–18). A key challenge in these studies is accounting for natural variation across sex and age. Age is the primary risk factor in OA (19–21), and both RA and OA are more prevalent in women (22, 23). Thus, investigating macrophage heterogeneity throughout life in both sexes is critical to better understanding the development and progression of these diseases.

Aging macrophages exhibit impaired function, increased inflammatory profiles, and have been implicated in a wide variety of age-associated diseases (24–26). With age, macrophages become less effective at key functions including phagocytosis, metabolism, antigen presentation, and clearance of senescent cells (27–32). Moreover, macrophages play a key role in “inflammaging,” the chronic low-grade inflammation associated with aging (33, 34). Macrophages mediate inflammaging through the production of pro-inflammatory cytokines, release of destructive enzymes, and recruitment of inflammatory cells (24, 35). A key characteristic of inflammaging is the skewed production of myeloid cells, particularly monocytes, in the bone marrow and spleen (36–40). Myeloid skewing, in combination with premature exit from the bone marrow, leads to increased numbers of immature circulating monocytes (30, 31, 41, 42). We have shown previously that aged monocytes are distinct from young monocytes on the transcriptional and epigenomic level (43). However, more investigation is needed to understand how aged monocytes, which may differentiate into macrophages in the tissue, influence macrophage composition in the joint.

In general, tissue macrophages derive from either embryonic precursors or monocytes as part of adult hematopoiesis (44–49). Over time, embryonically derived macrophages may be replaced by monocyte-derived macrophages that are functionally similar (50–57). Regardless of their origin, macrophages are capable of adapting to signal in the local environment through the combinatorial activity of cell-type and tissue-specific transcription factors (58, 59). In the joints of young healthy adult mice, expression of MHC-II distinguishes monocyte-derived macrophages from long-lived tissue-resident macrophages (10). Moreover, Culemann et al. demonstrated that CX3CR1 marks the synovial lining macrophages at steady-state with CX3CR1- macrophages localized to the interstitium or sublining (8). However, the composition of synovial macrophages changes drastically in inflammatory conditions with an influx of infiltrating monocytes (8, 10, 60). Macrophages derived from these pro-inflammatory monocytes are distinct from those that differentiate at steady-state (10, 52, 61, 62). Aging macrophages in other tissues demonstrate an increased proportion of monocyte-derived macrophages (54, 63). To our knowledge, no study has investigated synovial macrophage heterogeneity in aging.

Here, we use single-cell RNA-sequencing (scRNA-seq) to profile CD64+ synovial macrophages from the joints of young and old mice of both sexes. We define five major macrophage subpopulations and compare their proportions with aging in male and female mice. We also perform differential expression analysis to identify age-associated transcriptional changes. Finally, we infer differentiation trajectories and compare the density of samples across pseudotime based on age and sex. Our results reveal the sex-associated changes to synovial macrophages in the aging joint and lay the foundation for future studies of aging macrophages.

## Methods

### Mice

Male and female C56BL/6 mice were euthanized at approximately 8 (young) or 22 months (old) for this study (n=5 per sex/age). Mice were purchased from JAX and aged in house by the Bowdish Lab in pathogen-free conditions under the Institutional Animal Utilization protocols approved by McMaster University. At McMaster, mice were provided with nestlets, huts, and running wheels and fed Teklad aging diet with a 12-hr light cycle. Mice were shipped to Northwestern University and allowed to recover in quarantine for 4 months prior to euthanasia. Euthanasia was carried out via CO2 asphyxiation at 30-70% chamber volume per minute followed by cervical dislocation.

### Isolation of synovial macrophages

Both ankles from mice were dissected and skin was removed. Ankles were perfused with 3mL of digestion buffer (dispase II, collagenase D, and DNase I in 1xHBSS), and the synovium was exposed by cutting along the Achilles tendon into the joint space. The ankles were incubated in 1mL of additional digestion buffer for one hour at 37 °C in an orbital shaker. Post-digestion, the ankles and digestion buffer were filtered using a 40μm nylon mesh filter twice, and remaining synovial tissue was agitated against the mesh for optimal cell recovery. Erythrocyte lysis was performed using 1x PharmLyse and the single-cell suspensions were pooled by sex/age group to maximize cell recovery and stained for flow cytometry, followed by staining with viability dye, surface antibodies, and antibody-derived tags (ADT). Macrophages were isolated by fluorescence-activated cell sorting (FACS) based on a gating strategy for live CD45+CD11B+CD4/8/19/NK1.1-Ly6G-SiglecF-CD64+ as described previously (64) (**Supplementary Table 1**; **Supplementary Figure 1**). Identical gates were applied to each sample group. To mitigate batch effects, female samples (young and old) were processed together in parallel on the same day, and male samples (young and old) were processed together the following day. Due to animal death and mice removed from studies for health, the number of female samples pooled was reduced (n=4 for old, n=3 for young).

### Generation of single-cell libraries

Cellular Indexing of Transcriptome and Epitopes by sequencing (CITE-seq) was performed using the 10x Genomics 3’ GEX v3 and Feature Barcoding kits by the Metabolomics Core Facility (Genomics Services) at Northwestern University. BioLegend TotalSeq™-A mouse antibodies were used for profiling single-cell surface protein expression (**Supplementary Table 2**). Libraries were sequenced on an Illumina NextSeq 2000 targeting 40,000 reads per cell. The *cellranger v.7.1.0* pipeline was run with the mm10-2020-A reference transcriptome.

### Quality control

Gene expression counts were analyzed using Seurat v5.2.1 (64). Cells with low gene diversity (<200 unique genes) or low unique molecular identifier counts (<5000) were removed from consideration (**Supplementary Figures 2A-C**). Cells were filtered if their proportion of reads mapped to mitochondrial DNA exceeded 0.05 and their low-quality posterior probability was greater than 0.75 according to the bivariate regression models implemented in the miQC package (65). Doublets were predicted using scDblFinder (66) and removed. Summary cell numbers and quality control metrics can be found in **Supplementary Table 3**.

### Cell type annotation

Single-cell gene expression profiles were normalized and merged on 2,000 variable features using Seurat’s SCTransform procedure (67) with v2 regularization (68). Uniform manifold approximation and projection (UMAP) (69) embeddings were calculated on the top 20 PCs of the transformed variable genes with n=35 neighbors. Cell clustering was performed using Seurat’s nearest-neighbor clustering with k=20 on the top 20 PCs with 0.4 resolution. Cells were annotated according to the expression of cluster-specific gene markers, and highly correlated clusters were grouped (**Supplementary Figures 2D-G**). Macrophage cell clusters were retained for downstream analyses.

### Statistical analyses

Statistical differences in cell type proportions between groups was assessed using scProportionTest (70) with 10,000 permutations and false discovery rate (FDR) correction. Intercellular heterogeneity was measured as the average Euclidean distance from each cell’s coordinates in PC space (n=20) to the PC centroid of all cells for a given sample. To derive transcriptional divergence, pseudobulk expression aggregates were first normalized by downsampling without replacement to a shared mapping depth of 1 million reads, and then transcriptional divergence was calculated as the ratio between the mean expression of the top half and lower half of genes ranked by total expression (*https://github.com/DRWinterisCoding/DivergenceCalculator*). Differential gene expression was calculated using the FindMarkers() function in Seurat with the Wilcoxon rank-sum test on log-normalized RNA counts for genes expressed in at least 5% of cells by sample group and cell type, with FDR correction. Mitochondrial and ribosomal genes were excluded from consideration, as well as genes associated with rRNA contamination (71): *Gm42418*, *Malat1*, *Gm26917*, and *AY036118*. Genes with an adjusted P<0.05 and an average absolute log_2_ fold change (FC) >0.25 were considered significantly differentially expressed. Pathway enrichment was calculated for 1,730 canonical pathway gene sets from the Human Molecular Signatures Database (72) with 10,000 permutations on pathways with at least 10 genes ranked on relative gene expression changes between ages:


$$Weight=\;-\log_{10}\left(p\right)*log_{2}\left(FC\right)$$

Gene set variation analysis (GSVA) was applied at the single-cell level to estimate pathway-level activity in individual cells (73). Pseudotime trajectories among myeloid cells were derived using Monocle3 with ncenter=500 (74). Cell densities along pseudotime were estimated using kernel density estimation, and gene expression trajectories along pseudotime were modeled using generalized additive models (GAMs) with spline-based smoothing, as implemented in Monocle3 (74).

## Results

### Synovial macrophages consist of five major subpopulations

**Figure 1:**
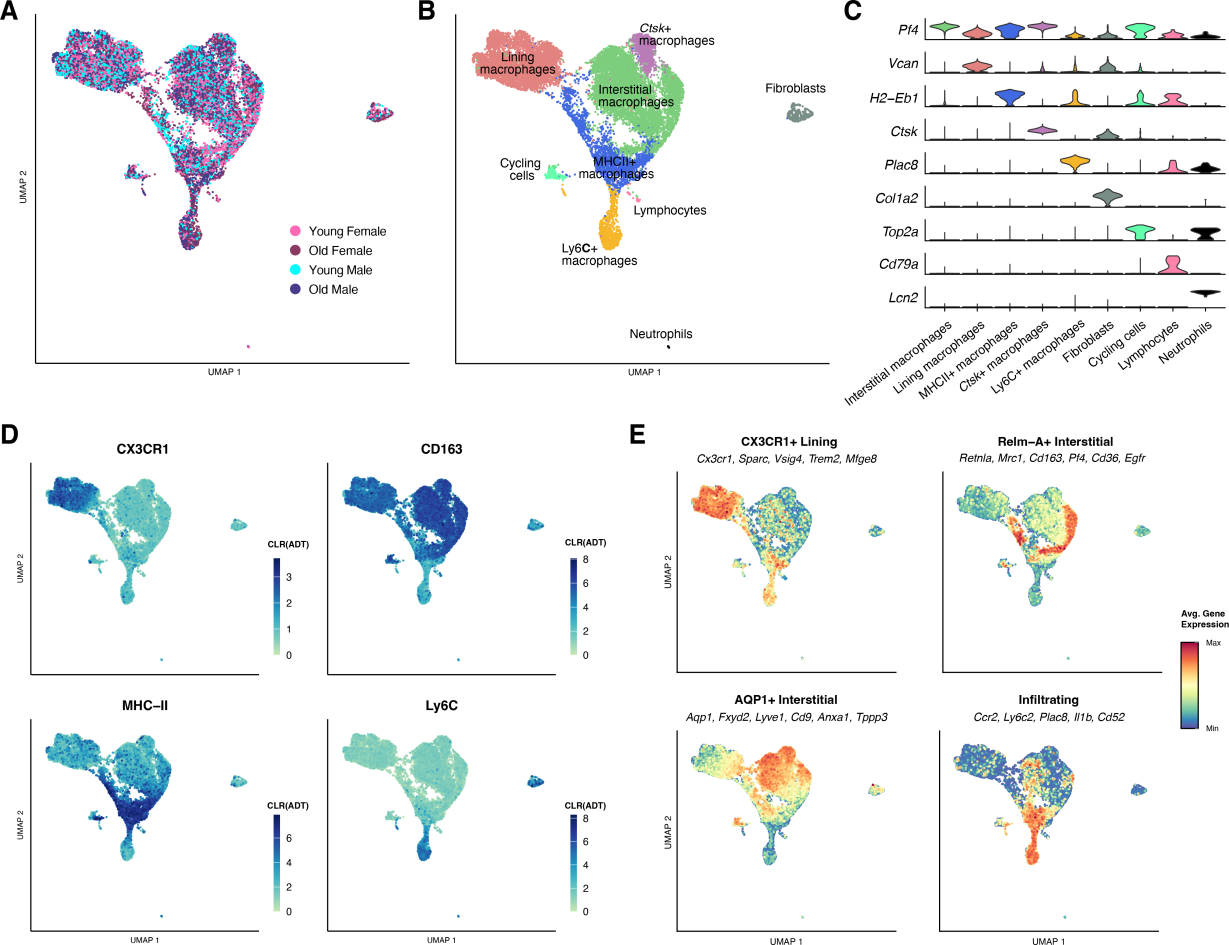
CITE-seq sample composition. **(A)** Integrated UMAP embedding of all cells (n=16,316) labeled by age and sex. **(B)** Integrated UMAP embedding of all cells labeled by cell type. **(C)** Expression by cell type of canonical markers. **(D)** Integrated UMAP embedding of all cells colored by surface protein expression. **(E)** Relative expression of cluster-specific gene signatures in integrated UMAP embedding.

To characterize synovial macrophage heterogeneity in aging, we performed CITE-seq, combining single-cell RNA-seq with cell surface marker levels, on macrophages isolated from the young and old joints of male and female mice. A total of 16,316 cells (4,019 mean cells per sample group) were retained for cell clustering and gene expression analysis (**Figure 1A**; **Supplementary Table 3**). Nearly all sampled cells were macrophage lineage (n=15,918; 97.56%), followed by fibroblasts (n=324; 1.99%), lymphoid cells (n=49; 0.30%);, and neutrophils (n=25; 0.15%) (**Supplementary Figures 2D-H**). Macrophages were annotated according to RNA and ADT expression of established cell-type-specific markers (**Figures 1B-E**; **Supplementary Table 4**). We identified five major synovial macrophage subpopulations: Ly6C+ infiltrating macrophages, MHCII+ monocyte-derived macrophages, CX3CR1+ lining macrophages, CD163+ interstitial macrophages, and *Ctsk*-expressing macrophages (*Ctsk*+), as well as cycling cells (**Figure 1B**). Although the *Ctsk+* subpopulation was also CD163+, the unique expression of *Ctsk*, a marker of osteoclast lineage, suggested these cells should be analyzed independently (**Figures 1B-D**). At a higher resolution, we identified distinct cell clusters of *Arg1*-expressing (*Arg1*+) or non-expressing (*Arg1*-) cells within the MHCII+ subpopulation and *Aqp1*, *Clec10a*, or *Retnla*-expressing cells within the Interstitial subpopulation (**Supplementary Figures 2D, E**). These annotations were supported by the expression of genes identified in Culemann et al. (8) and consistent with fate-mapping studies by Misharin et al. (10).

### Age-associated differences in synovial macrophage composition are sex-dependent

**Figure 2:**
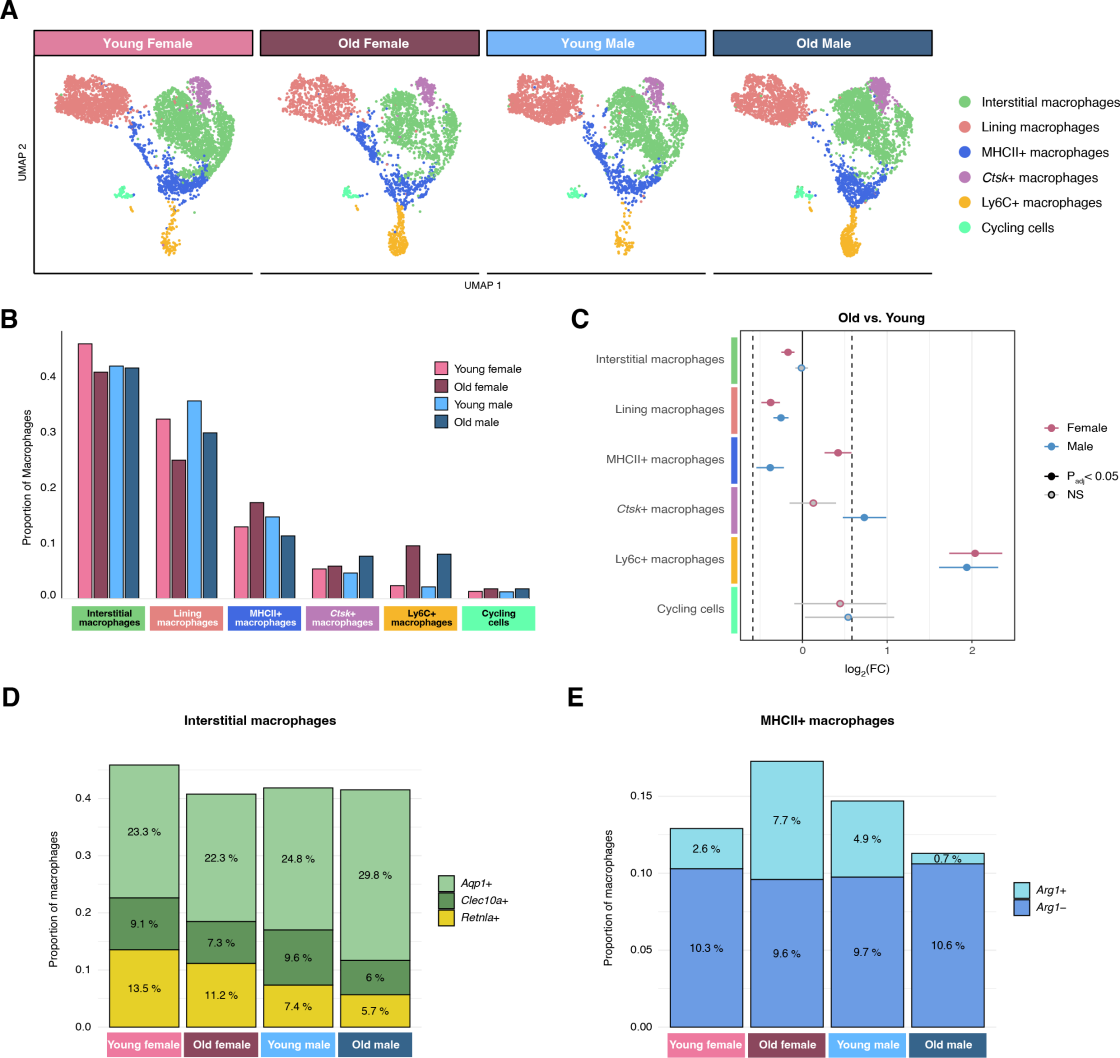
Landscape of synovial macrophages. **(A)** Integrated UMAP embedding of synovial macrophage subpopulations (n=15,918) split by age and sex. **(B)** Proportions of macrophage subpopulations by age and sex. **(C)** Forest plot of bootstrapped pairwise differences in macrophage subpopulation proportions in old vs. young mice. NS, not significant. **(D)** Composition of interstitial macrophages in terms of *Aqp1*, *Clec10a*, and *Retnla*-expressing cells, by age and sex. **(E)** Composition of MHCII+ macrophages in terms of *Arg1*-expressing cells.

In order to identify changes in synovial macrophage composition with age, we compared the proportions of subpopulations between young and old mice. We observed significant differences in aging synovial macrophage proportions across sexes (**Figures 2A-C**). The most pronounced difference with age was a significant expansion of infiltrating Ly6C+ macrophages in both males and females (8.0% vs. 2.1%, and 9.5% vs. 2.3%, respectively; p<0.0001) which is consistent with an increased contribution of monocytes in aging. We also observed significant reductions in the proportion of CX3CR1+ lining macrophages with age in both sexes (35.6% vs. 29.8% in males, and 32.4% vs. 24.9% in females, p<0.0001). These proportional changes aligned with those observed via flow cytometry (**Supplementary Figures 1D, E**). Other changes in subpopulation proportions were unique to either sex. *Ctsk*+ macrophages were significantly increased with age in males (7.6% vs. 4.6%, p<0.0001) but not in females (5.8% vs. 5.3%, p=0.2). Interstitial macrophages were significantly reduced with age in females (40.8% vs. 45.9%, p<0.0001), with proportional reductions in *Aqp1*, *Clec10a*, and *Retnla*-expressing cells (**Figure 2D**; **Supplementary Figures 3A, B**). In males, the proportion of interstitial macrophages did not change with age (41.5% vs. 41.9%, p=0.4), despite a notable increase in *Aqp1*-expressing cells and corresponding reductions in *Clec10a* and *Retnla*-expressing cells. Interestingly, there were significantly more MHCII+ macrophages in aged female mice (17.3% vs. 12.9%, p<0.0001), but significantly fewer in aged male mice (11.3% vs. 14.7%, p<0.0001). The contrasting changes in the proportion of MHCII+ macrophages by sex were driven by *Arg1*-expressing cells, which were expanded in aged females but were drastically reduced in aged males (**Figure 2E**).

The intercellular heterogeneity of the synovial macrophages, defined as a sample’s average cell-to-centroid distance in PCA space, was significantly greater in aged vs. young mice in females (p<0.0001), but not statistically different in males (**Supplementary Figure 3C**). Transcriptional divergence, i.e. the degree of expression difference between highly and lowly expressed genes, among synovial macrophages was not statistically different between any sample groups (**Supplementary Figure 3D**). Overall, we observed differences in macrophage subpopulation proportions with age that were consistent between both sexes, most notably for Ly6C+ cells, as well as sex-specific effects, such as the contrasting changes in MHCII+ macrophage prevalence.

### Upregulation of electron transport pathway and downregulation of MAPK signaling with aging

**Figure 3:**
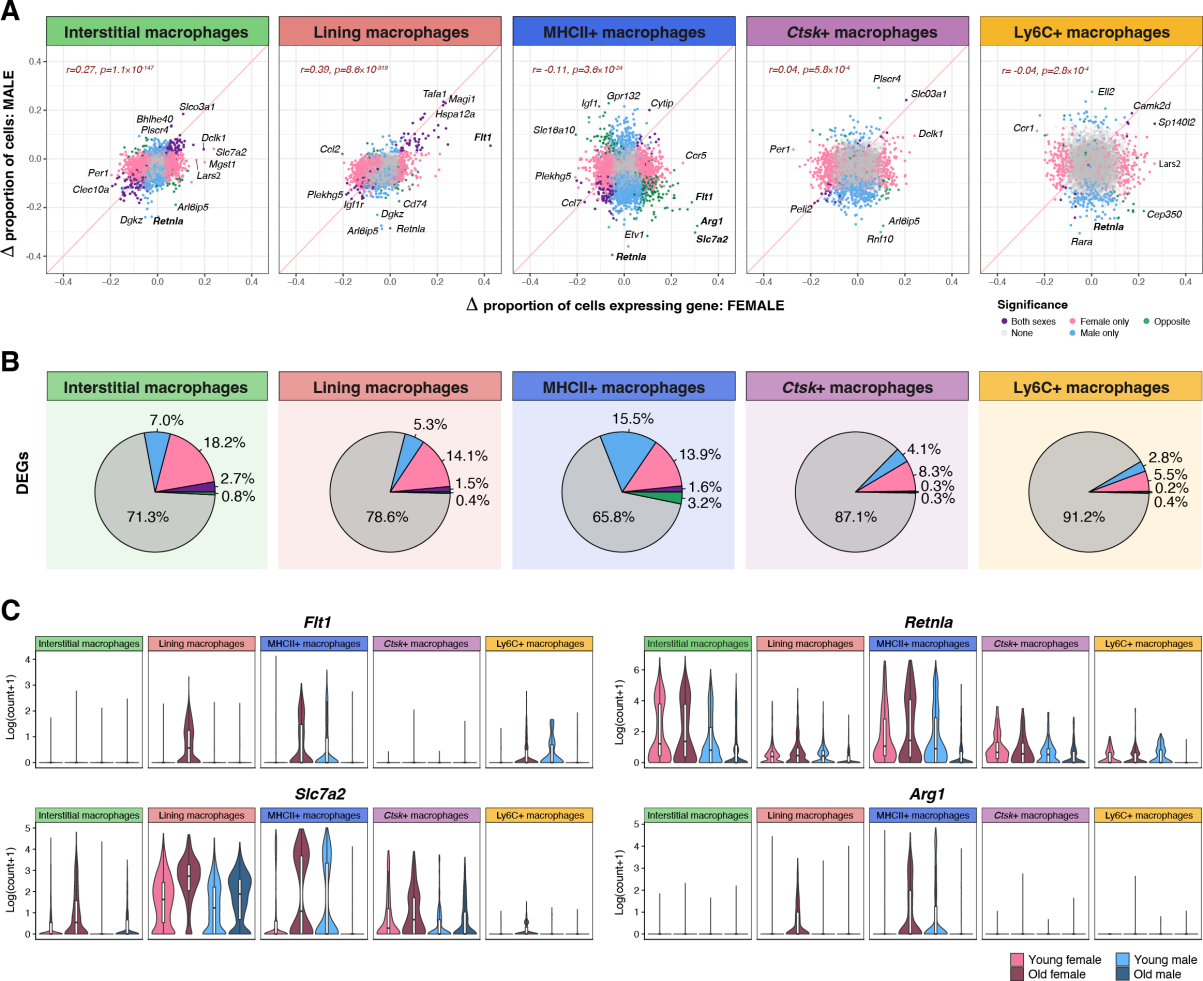
Differential gene expression between groups in synovial macrophages. **(A)** Changes in the proportion of macrophage subpopulations expressing each gene in old mice vs. young mice are shown for males (Y axis) against those in females (X axis) for each subpopulation. Significant DEGs in both conditions are highlighted in blue, significant DEGs in males only are highlighted in blue, and significant DEGs in females only are highlighted in pink. Significant DEGs with opposite directions of effect between sexes are highlighted in green. The correlations between comparisons are shown in the upper left. The diagonal pink lines denote y=x. Select genes are labeled. **(B)** Proportions of genes that were significantly differentially expressed by age, split by sex and direction of effect. **(C)** Distributions of select gene expression by age, sex, and macrophage subpopulation.

Next, we compared the transcriptional profiles of major synovial macrophage populations across aging by sex. We found that differential gene expression with age was most prominent in the interstitial and lining macrophage subpopulations (**Figures 3A, B**; **Supplementary Table 5**), though this may be at least partially due to the increased power offered by their greater cell numbers. Many of the differentially expressed genes (DEGs) were shared across these subpopulations (**Supplementary Figures 4A, B**). Differential expression was modestly correlated between sexes in interstitial (r=0.27 for percentage of cells expressing each gene, p=7.3×10^-147^) and lining macrophages (r=0.39, p=8.6×10^-319^), but was less concordant in the other subpopulations. The MHCII+ subpopulation, in particular, featured a negative overall correlation in gene expression changes between sexes (r= -0.11, p=3.6×10^-24^), led by discordant expression changes in *Arg1*, *Slc7a2*, and *Retnla* genes (**Figure 3C**).

**Figure 4:**
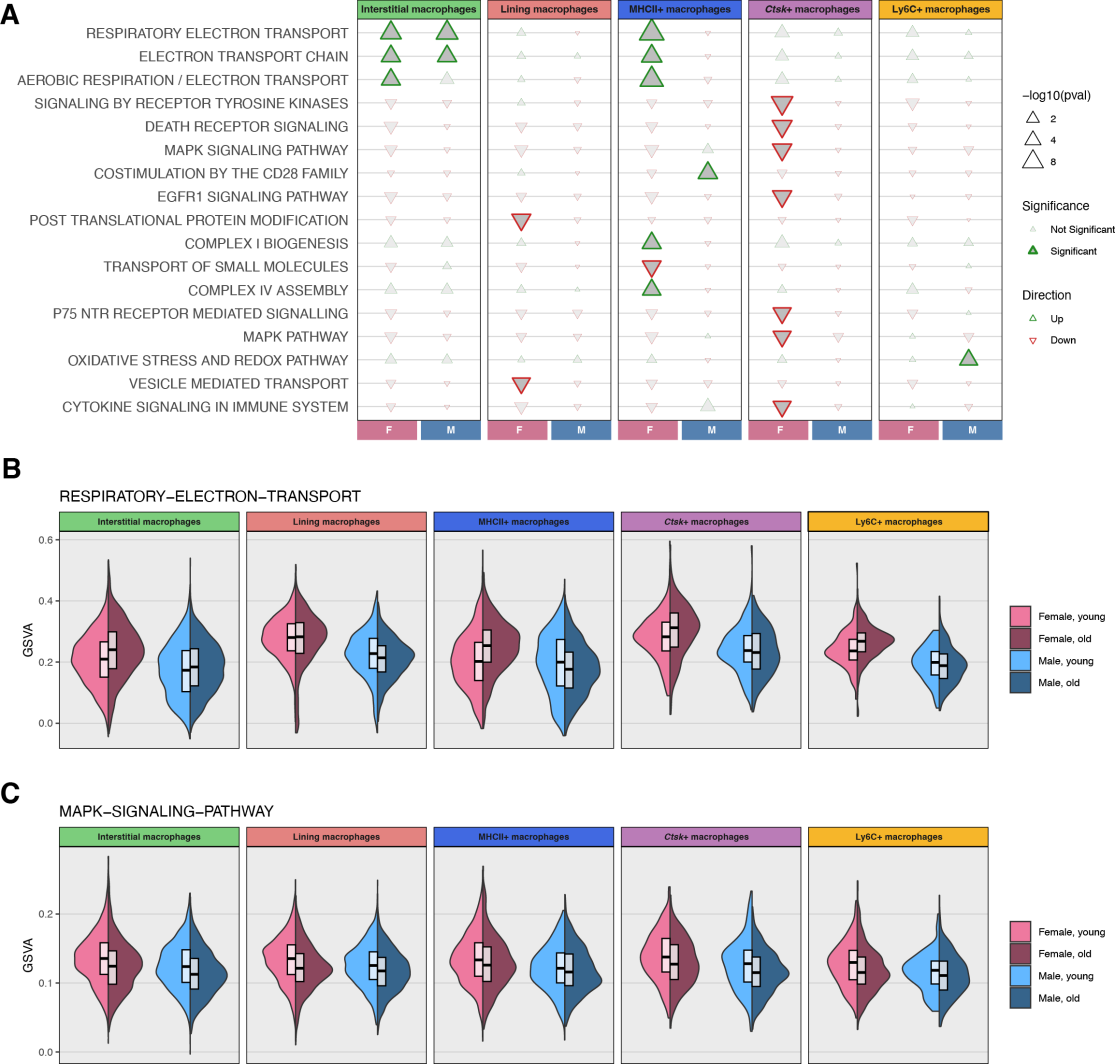
Pathway enrichment in aging synovial macrophages. **(A)** Pathways significantly enriched by age in at least one macrophage subpopulation, split by sex. Upregulated pathways are shown in green, downregulated in red. **(B)** Distribution of Gene Set Variation Analysis (GSVA) scores for Respiratory Electron Transport genes, reflecting the relative activity of the pathway across cells (higher scores indicate greater coordinated expression of pathway genes), by age, sex, and macrophage subpopulation. **(C)** Single-cell GSVA scores for the MAPK Signaling Pathway by age, sex, and macrophage subpopulation.

Age-associated gene expression changes were significantly enriched for a variety of biological pathways (**Figure 4A**; **Supplementary Table 6**). Genes associated with electron transport chain signaling were significantly upregulated in interstitial macrophages in both sexes and in MHCII+ macrophages in females (**Figure 4B**). The enrichment signal was driven primarily by increased expression of Complex I and Complex IV genes (**Supplementary Table 6**). Several other pathways trended down in most subpopulations but were only significantly enriched among downregulated genes in female *Ctsk*+ macrophages, including receptor tyrosine kinase, death receptor, MAPK (**Figure 4C**), EGFR, and immune cytokine signaling pathways (**Supplementary Figure 5**), which is interesting as these cells were only significantly expanded in males. Common leading-edge genes across a majority of these pathways included *Egfr*, *Mapk1*, *Map2k2*, *Mapk14*, *Nfkb1* (**Supplementary Figure 4C**)*, Tnf* (**Supplementary Figure 4D**)*, Prkcb*, and *Vav1* (**Supplementary Table 6**). Among infiltrating Ly6C+ macrophages, oxidative stress genes were significantly upregulated only in males. While the changes to individual pathways may be subtle, the general concordance across subpopulations suggests widespread changes with age.

### Synovial macrophage differentiation features several distinct trajectories

**Figure 5:**
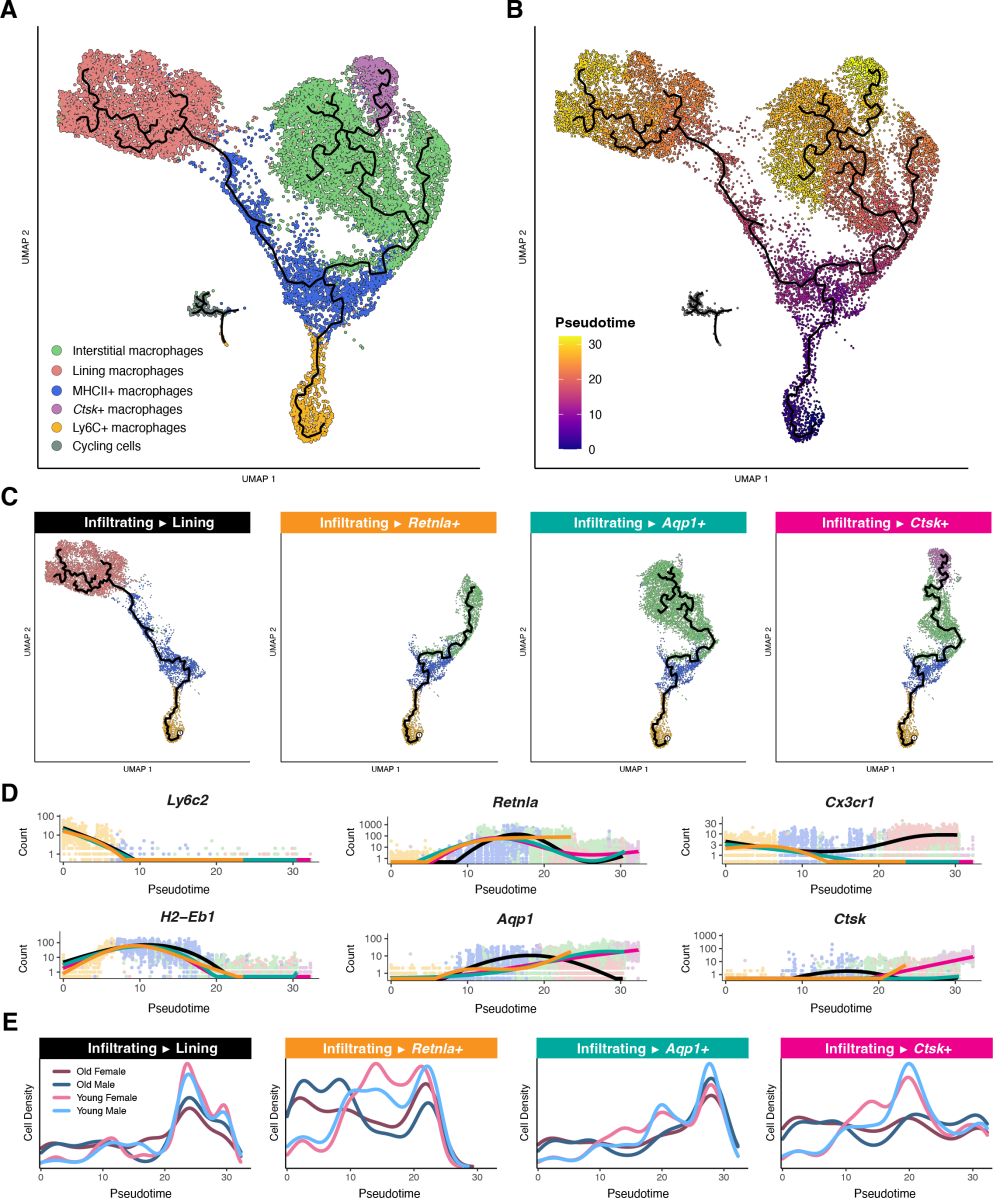
Pseudotemporal distribution of synovial macrophages. **(A)** Integrated UMAP embedding of synovial macrophage subpopulations with trajectories inferred by pseudotime overlaid in black. **(B)** Integrated UMAP embedding of synovial macrophages colored by pseudotime distance from the root infiltrating Ly6C+ macrophages. **(C)** Distinct pseudotime branches from root infiltrating Ly6C+ macrophages to each of four different terminal subpopulations **(D)** Gene expression by pseudotime for key subpopulation genes. Expression counts are shown for each cell, colored by macrophage subpopulation, and modeled for each distinct pseudotime branch with spline curves. **(E)** Cell density vs. pseudotime modeled for each pseudotime branch, split by age and sex.

To map the differentiation paths of infiltrating macrophages into synovial tissue, we fit pseudotime trajectories to the transcriptional and cell-surface protein data rooted in Ly6C+ macrophages (**Figures 5A, B**; **Supplementary Figure 6**). The pseudotime modeling revealed four trajectories terminating in different macrophage subpopulations and *Ctsk*+ macrophages (**Figure 5C**). Upon infiltration into the synovium, *Ly6c2* expression in all macrophages decreases and *H2-Eb1* expression transiently increases (**Figure 5D**). Within MHCII+ macrophages, differentiation bifurcates into a *Cx3cr1*-expressing terminus of lining macrophages versus the interstitial and *Ctsk*+ populations, marked by *Retnla*, *Aqp1*, and *Ctsk* expression, respectively. In the aged synovial niche from both male and female mice, we observe increased density of macrophages at the early pseudotime-points and a reduction at key transitional points across the different trajectories, including an apparent reduction in differentiation from infiltrating macrophages into *Retnla*-expressing cells with age in both sexes (**Figure 5E**). This analysis provides insight on the possibility of impaired differentiation of macrophages in aging joints.

## Discussion

In this study, we use single-cell technology to investigate synovial macrophage heterogeneity in aging. We define five major macrophage subpopulations based on the literature. We observed age-associated differences in subpopulation proportions that are conserved and distinct between sexes. In particular, we found significantly increased proportions of Ly6C+ infiltrating macrophages and significantly decreased proportions of CX3CR1+ lining macrophages with aging in both males and females. On the other hand, MHCII+ macrophages, which have previously been shown to be monocyte-derived (10), varied in proportion in opposite directions between sexes and were associated with distinct transcriptional changes in aging. Aging synovial macrophages also exhibited differential expression of genes involved in specific biological pathways, such as electron transport chain and MAPK signaling. These datasets are available for further exploration through our hosted UCSC Cell Browser (75) at https://winterlab-cellbrowser.fsm.northwestern.edu/Cell-Browser_Aging_Joint_Synovial_Macrophages/. Taken together, our results support a shift in the synovial macrophage compartment in aging that is sex-dependent.

As expected, we find an increased number of Ly6C+ macrophages, which we classify as infiltrating based on their expression of monocyte genes. Despite their transcriptional similarity to monocytes, these cells were part of the CD64+ fraction of synovial myeloid cells. In our prior work, we have shown that there is a distinct population of CD64- synovial myeloid cells of which a proportion are very similar to circulating monocytes and are positively labelled by intravascular staining (76). However, this population is not included in the current analysis. Therefore, we infer that these Ly6C+ cells are newly arrived monocyte-derived cells distinct from the MHCII+ macrophages, which have likely been in residence for a longer period (10). The expansion of infiltrating macrophages in aged joints is consistent with prior work showing a greater proportion of monocyte-derived macrophages in other aged tissues (54, 63). The persistence of monocyte gene expression may suggest an increased rate of infiltration rather than simply an accumulation over time. Alternatively, it may reflect an impaired differentiation trajectory that prevents these cells from adopting a more tissue-resident phenotype. These possibilities are not mutually exclusive and may reflect aspects of the chronic low-grade inflammation associated with aging.

Sex-associated differences have previously been noted in the transcriptional profile of tissue macrophages and may be exacerbated with age. Prior work from our group noted that biological sex, rather than reproductive cycle, was responsible for differences observed in mice, such as fewer monocytes observed in females (77). In general, macrophages are thought to have higher activation and phagocytic capacity in females with higher TLR4 and pro-inflammatory cytokine expression in males (78). In a meta-analysis, the Benayoun group found that macrophages across tissues exhibited sex-dimorphic gene regulation though the relevant pathways varied across niches (79). In particular, microglia, the brain-resident macrophages, were demonstrated to have differential expression between sexes that develops in adults (80, 81). Sex-specific microglia expression was further augmented with aging, leading to differential phenotypes (82). Similarly, peritoneal macrophages have been shown to deviate between sexes with age, partially due to hormones (83). In data from our previous study, we found differences in the proportions of synovial macrophages in steady-state and over the course of a joint trauma model (60). Thus, our results demonstrating sex-associated changes in synovial macrophages with ages are consistent with literature findings. Further investigation may connect these results to differential penetrance between sexes in mouse models of inflammatory arthritis and possibly to sex-dependent presentation of disease in patients.

Our initial annotation of macrophage subpopulations included the identification of a cluster we labelled as *Ctsk*+ macrophages. *Ctsk* (Cathepsin K) is a well-established marker of osteoclast lineage, suggesting these cells may represent osteoclast precursors, which have been shown to arise from the monocyte/macrophage lineage (84). Eventually, they will differentiate into multinucleated osteoclasts that break down bone tissue (85). While this process is a natural part of bone growth and repair, abnormally activated osteoclasts contribute to the pathology of arthritis (86). Several studies have identified similar subpopulations or transcriptional signatures that suggest the presence of osteoclast precursors, or “osteoclast-like” cells (87), among the synovial macrophage compartment, especially in inflammatory arthritis (8, 60, 88). In our study, the *Ctsk*+ population was significantly expanded with age in males but not in females. Furthermore, older age in females coincided with a significant downregulation of MAPK and cytokine signaling in these cells, which are required for osteoclast differentiation (89). In OA, females exhibit lower percentages of mature osteoclasts compared to males in OA (90). Therefore, our findings suggest that *Ctsk*-expressing macrophages contribute to sex differences in age-associated arthritis.

Pseudotime trajectory analysis revealed age-associated differences in macrophage differentiation dynamics. Ly6C+ infiltrating macrophages were designated as the trajectory root based on studies demonstrating Ly6C+ monocytes can serve as precursors for tissue-resident macrophages (50, 91) and consistent with prior synovial macrophage analyses (87). Importantly, pseudotime analyses capture transcriptional relationships and may not represent true developmental lineages. Therefore, the inferred infiltrating-to-lining trajectory may reflect transcriptional similarity rather than direct differentiation, since synovial lining macrophages have been found to be predominantly embryonically derived (92). In aged mice, we observed a reduced density of differentiation into RELM-α+ (*Retnla*+) macrophages in both sexes and lower proportions of these cells among interstitial macrophages This age-related deficit in homeostatic macrophages (93, 94) could contribute to the persistence of low-grade inflammation and increased susceptibility to joint pathology with aging.

The current study is limited by several factors related to the single-cell technology and experimental design. First of all, it is not possible to ascertain changes in the absolute numbers of synovial macrophage subpopulations since the single-cell datasets represent only samplings of the compartment. Even with flow cytometry data, it is difficult to account for relative changes in cell number due to increased immune infiltration and cell death as part of ankle processing. Additionally, enzymatic digestion may differentially affect recovery of macrophage subtypes; for example, more adherent or fragile populations could be under-represented in the final single-cell suspension. Moreover, variation in cell number across sample groups and subpopulations leads to ranges in statistical power for inferences about differential expression and cell compositions, and this affects our ability to determine which populations are the most altered in an objective manner. Finally, mice were pooled by sex/age group prior to sorting, which precludes assessment of inter-individual variation. Morbidity and mortality reduced the number of mice contributing to female pooled groups (n=3 young, n=4 old vs. n=5 per male group), which may have had an impact on the comparable statistical power. Future studies with larger sample sizes and preserved biological replicates would enable formal assessment of inter-individual variability and strengthen the generalizability of findings, particularly for sex-specific effects.

In summary, our study reveals the sex-associated changes in synovial macrophages from the aging joint. These results provide insight on interpreting aging macrophage studies in joints and other tissues when only one sex is presented. Our conclusions also indicate that additional research is required to dissect the contribution of monocyte-derived macrophages with aging. Studies with expanded time points that span the life of the mouse are needed. Furthermore, epigenomic studies that reveal the underlying changes in gene regulation and functional assays to understand the impact on phagocytic capacity, cytokine production, and mitochondrial respiration are key to identifying how age-related dysfunction arises. Parallel analysis of circulating monocytes and other macrophage populations would help distinguish local, joint-specific changes from systemic, age-related effects on myeloid cells. Continued research using mouse models to study cells *in vivo* and identify key factors will shed light on how age contributes to the development of arthritis and related diseases in humans.

## Data Availability

The datasets presented in this study can be found in online repositories. Count matrices for gene expression and antibody-derived tag (ADT) libraries from single-cell CITE-seq have been deposited in the Gene Expression Omnibus (GEO) under accession number GSE312430 (https://www.ncbi.nlm.nih.gov/geo/query/acc.cgi?acc=GSE312430). An interactive cell browser for exploring the processed single-cell data is available at https://winterlab-cellbrowser.fsm.northwestern.edu/Cell-Browser_Aging_Joint_Synovial_Macrophages/.

## Supplementary material

Supplementary Figure 1 Flow cytometry gating schema and cell counts. **(A)** Flow gating strategy for CD45+CD11B+ cells. Images are from the young female sample group. **(B)** Flow gating strategy for isolation of macrophages used in single-cell analysis (pink) and quantification of dendritic cells (DCs), granulocytes, and monocytes. Black arrows indicate positive gating, while red arrows indicate negative gating. Images are from the young female sample group. **(C)** Proportions of CD45+CD11B+ cell populations among live cells (left), and proportions of monocytes by Ly6C expression levels (right), by age and sex. **(D)** Flow gating strategy for identification of macrophage subpopulations. **(E)** Proportions of macrophage cell populations, by age and sex.

Supplementary Figure 2 Cell clustering and cell type annotation. **(A)** Violin plots showing the distributions of Unique Molecular Identifiers (UMIs) per cell, by age and sex. The dashed red line indicates 5,000 UMIs/cell, below which cells were removed during quality control. **(B)** Violin plots of unique features captured per cell, by age and sex. **(C)** Cellular distributions are shown by age and sex for the percentage of reads mapping to mitochondrial DNA. The dashed red line indicates 5% of reads. **(D)** Integrated UMAP of all cells following quality control, colored by cell type. **(E)** Heatmap of top cell type markers genes. **(F)** Composite lymphocyte gene expression among all cells. **(G)** Composite neutrophil gene expression among all cells. **(H)** Heatmap of gene expression correlations between cell types.

Supplementary Figure 3 Cell type distributions and heterogeneity. **(A)** Integrated UMAP of all cells following quality control, colored by cell type, split by age and sex. **(B)** Cell type proportions by age and sex. **(C)** Intercellular heterogeneity of synovial macrophages by age and sex. **(D)** Transcriptional divergence by age and sex.

Supplementary Figure 4 Differential gene expression counts in synovial macrophages. Upset plots of counts of significantly differentially expressed genes by age within each synovial macrophage subpopulation in **(A)** females and **(B)** males. **(C)** Distributions of *Nfkb1* expression by age, sex, and macrophage subpopulation. *FDR-adjusted p<0.05. **(D)** Distributions of *Tnf* expression by age, sex, and macrophage subpopulation. * FDR-adjusted p<0.05.

Supplementary Figure 5 Distributions of significantly enriched pathway genes. Changes in the proportion of macrophage subpopulations expressing each gene in old mice vs. young mice are shown for males (Y axis) against those in females (X axis) for each subpopulation in select enriched pathways. Leading edge genes are highlighted in dark blue, and other pathway genes are highlighted in pink. Highlighted quadrants depict significant enrichment among upregulated (green) and downregulated (red) genes for a given sex.

Supplementary Figure 6 Pseudotemporal expression of cell surface proteins. Normalized expression of antibody-derived tags (ADT) from CITE-seq are shown by pseudotime for key subpopulation genes. Expression is shown for each cell, colored by macrophage subpopulation, and modeled for each distinct pseudotime branch with spline curves.
