# Supplementary figures and images for: Sex-associated transcriptional changes to synovial macrophages in the aging joint
Source: Front Immunol. 2026 Mar 3;17:1724385. doi: 10.3389/fimmu.2026.1724385 (PMC12992061; doi:10.3389/fimmu.2026.1724385)

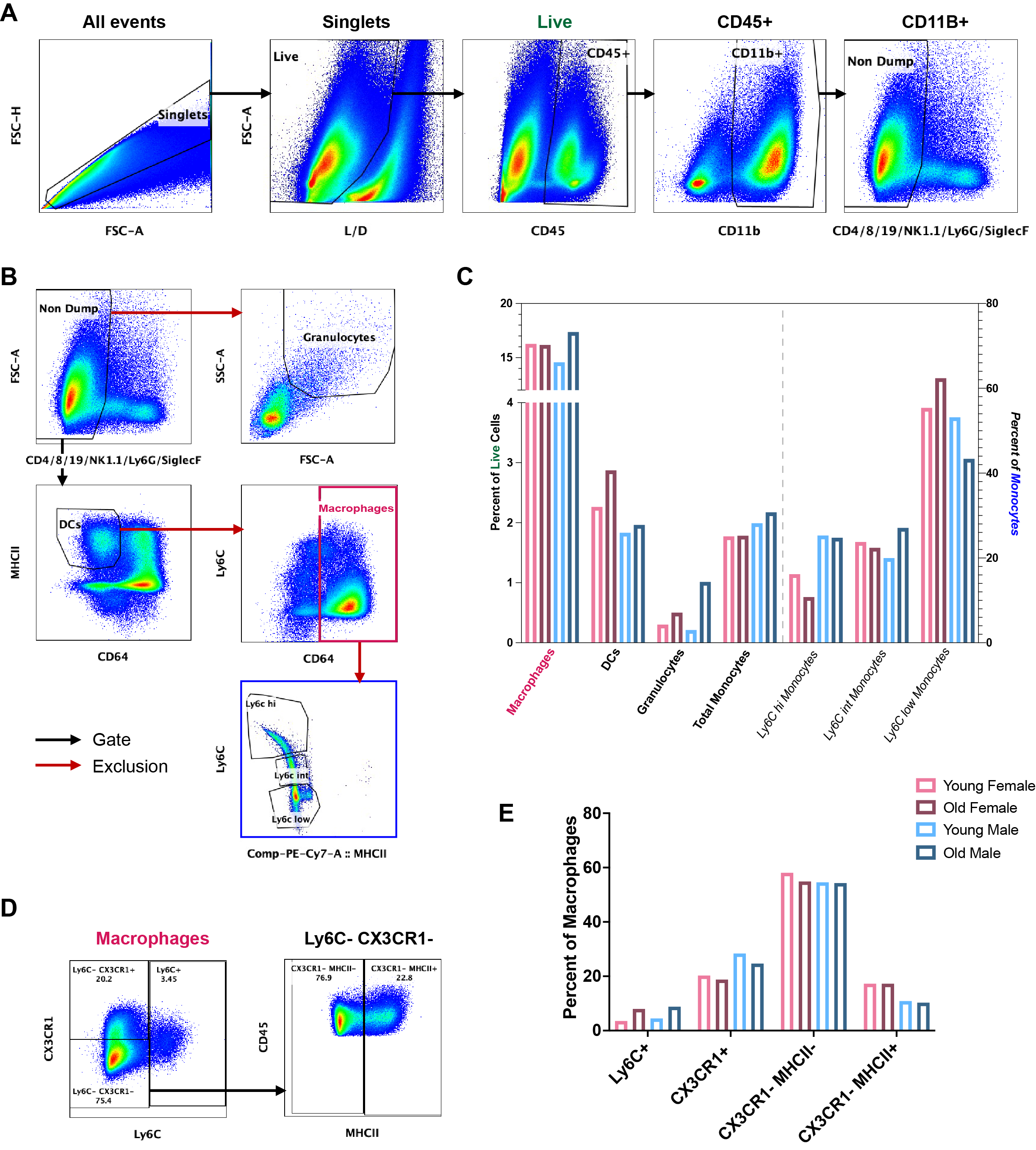

Supplement: Supplementary Figure 1 — Flow cytometry gating schema and cell counts. (A) Flow gating strategy for CD45+CD11B+ cells. Images are from the young female sample group. (B) Flow gating strategy for isolation of macrophages used in single-cell analysis (pink) and quantification of dendritic cells (DCs), granulocytes, and monocytes. Black arrows indicate positive gating, while red arrows indicate negative gating. Images are from the young female sample group. (C) Proportions of CD45+CD11B+ cell populations among live cells (left), and proportions of monocytes by Ly6C expression levels (right), by age and sex. (D) Flow gating strategy for identification of macrophage subpopulations. (E) Proportions of macrophage cell populations, by age and sex. [file Image1.tif]

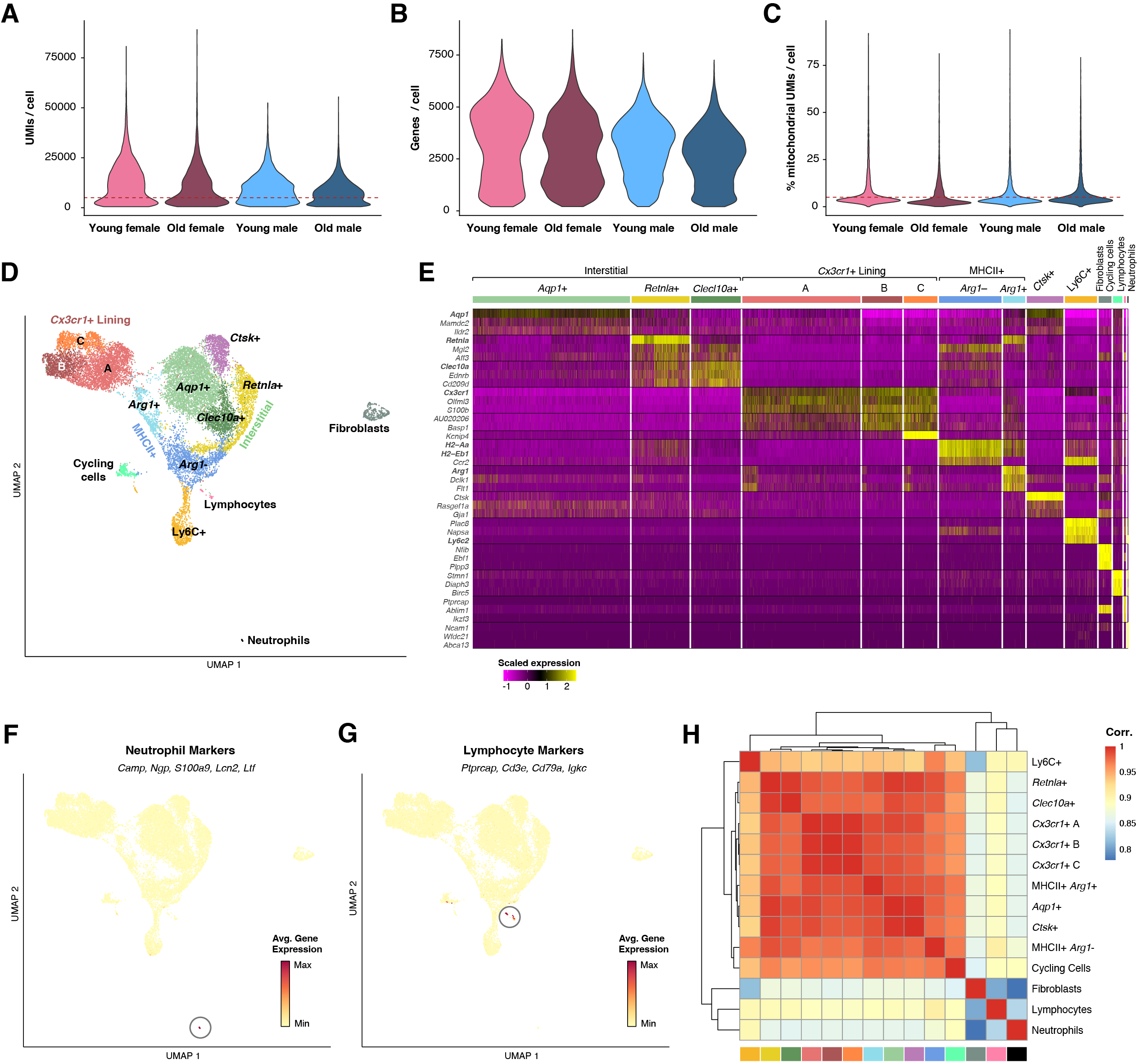

Supplement: Supplementary Figure 2 — Cell clustering and cell type annotation. (A) Violin plots showing the distributions of Unique Molecular Identifiers (UMIs) per cell, by age and sex. The dashed red line indicates 5,000 UMIs/cell, below which cells were removed during quality control. (B) Violin plots of unique features captured per cell, by age and sex. (C) Cellular distributions are shown by age and sex for the percentage of reads mapping to mitochondrial DNA. The dashed red line indicates 5% of reads. (D) Integrated UMAP of all cells following quality control, colored by cell type. (E) Heatmap of top cell type markers genes. (F) Composite lymphocyte gene expression among all cells. (G) Composite neutrophil gene expression among all cells. (H) Heatmap of gene expression correlations between cell types. [file Image2.tif]

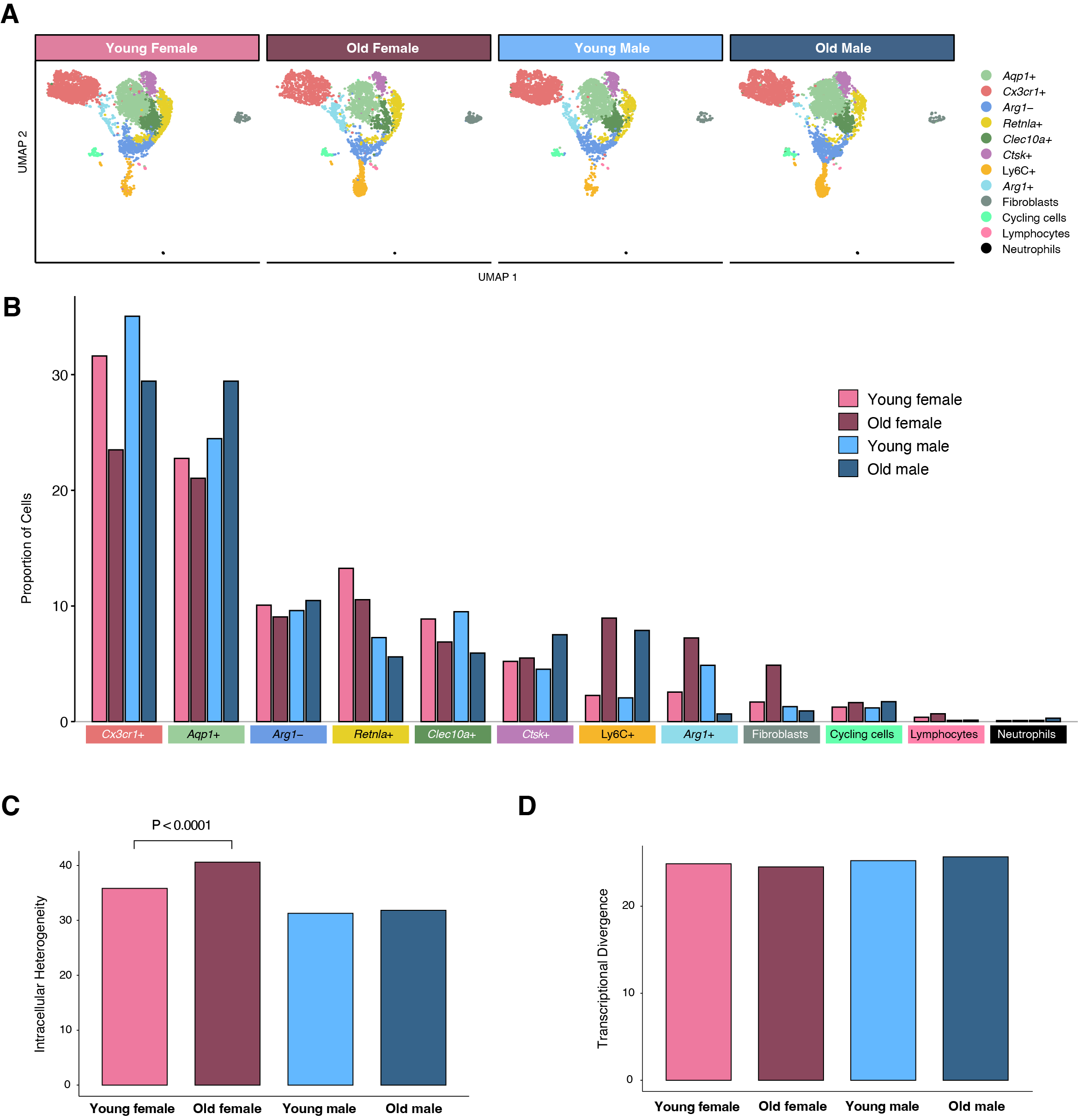

Supplement: Supplementary Figure 3 — Cell type distributions and heterogeneity. (A) Integrated UMAP of all cells following quality control, colored by cell type, split by age and sex. (B) Cell type proportions by age and sex. (C) Intercellular heterogeneity of synovial macrophages by age and sex. (D) Transcriptional divergence by age and sex. [file Image3.tif]

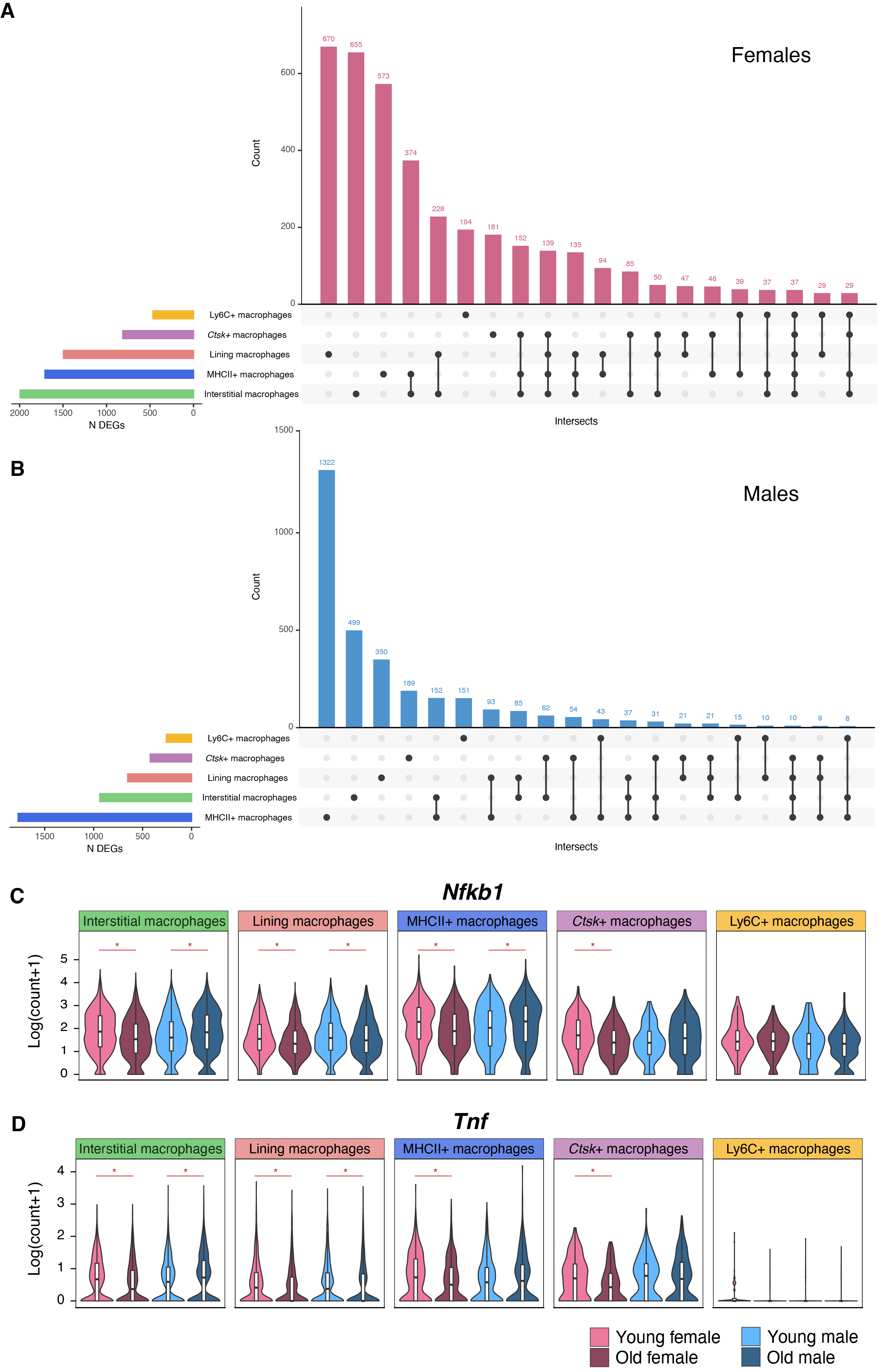

Supplement: Supplementary Figure 4 — Differential gene expression counts in synovial macrophages. Upset plots of counts of significantly differentially expressed genes by age within each synovial macrophage subpopulation in (A) females and (B) males. (C) Distributions of Nfkb1 expression by age, sex, and macrophage subpopulation. *FDR-adjusted p<0.05. (D) Distributions of Tnf expression by age, sex, and macrophage subpopulation. * FDR-adjusted p<0.05. [file Image4.tif]

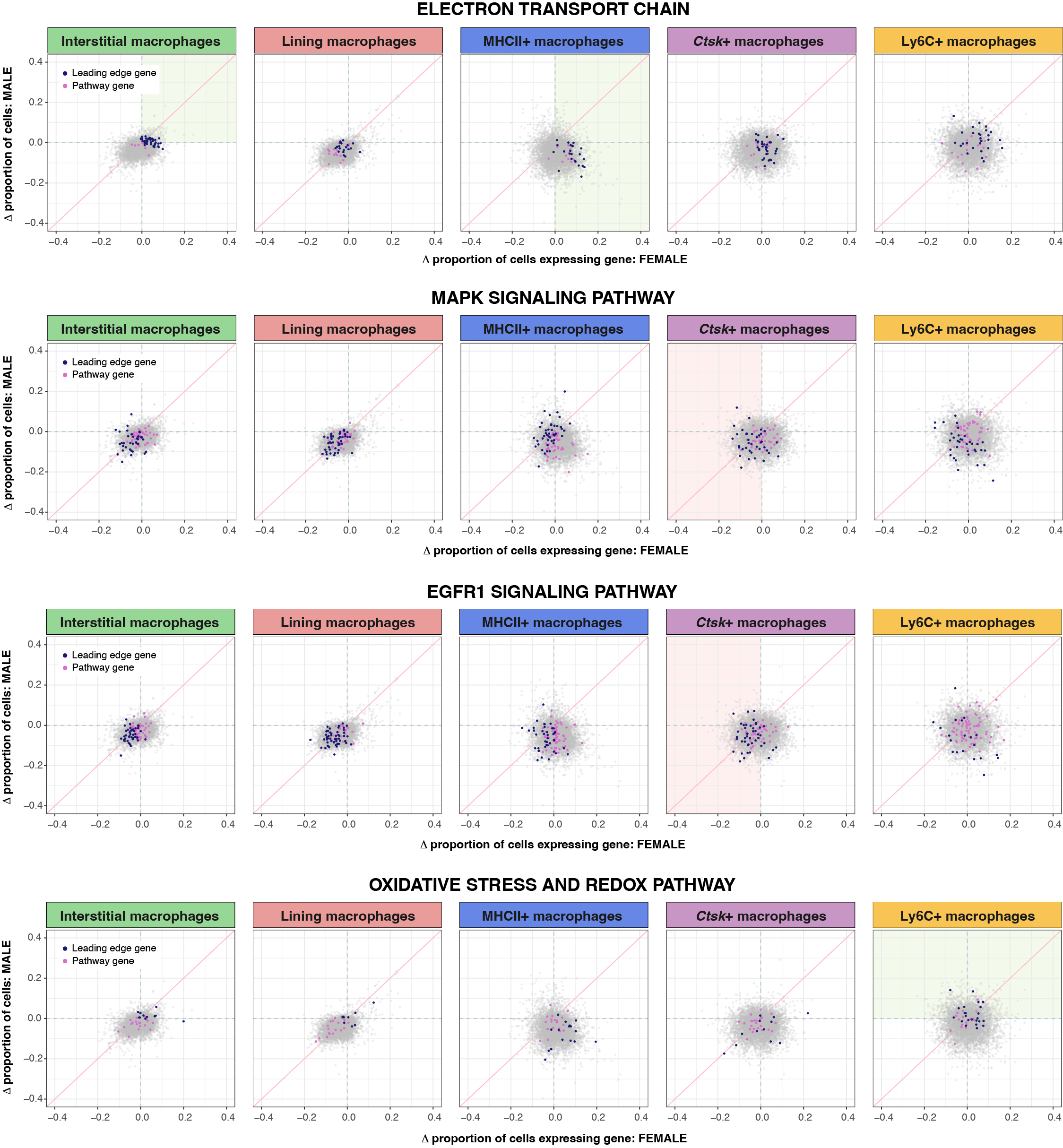

Supplement: Supplementary Figure 5 — Distributions of significantly enriched pathway genes. Changes in the proportion of macrophage subpopulations expressing each gene in old mice vs. young mice are shown for males (Y axis) against those in females (X axis) for each subpopulation in select enriched pathways. Leading edge genes are highlighted in dark blue, and other pathway genes are highlighted in pink. Highlighted quadrants depict significant enrichment among upregulated (green) and downregulated (red) genes for a given sex. [file Image5.tif]

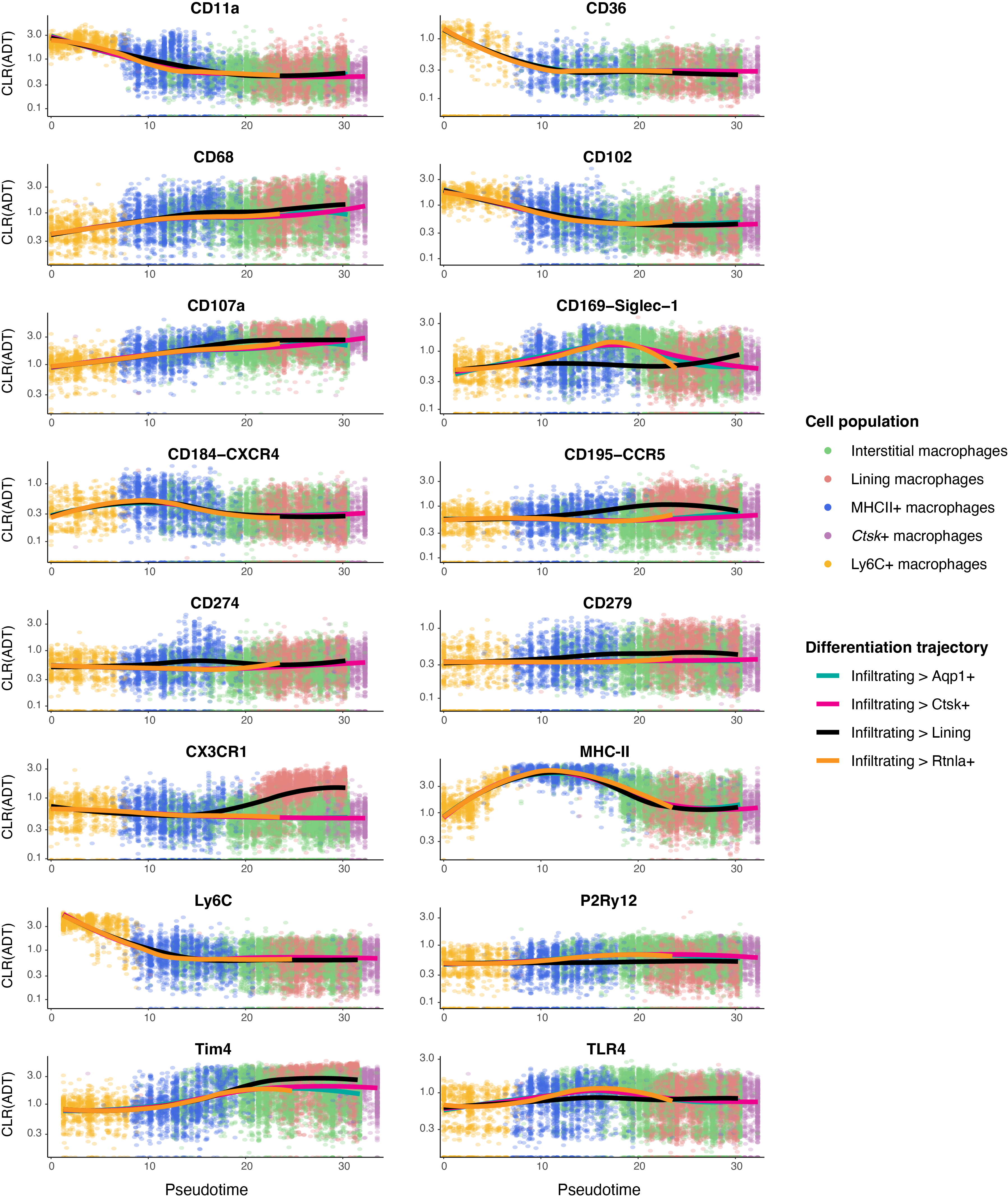

Supplement: Supplementary Figure 6 — Pseudotemporal expression of cell surface proteins. Normalized expression of antibody-derived tags (ADT) from CITE-seq are shown by pseudotime for key subpopulation genes. Expression is shown for each cell, colored by macrophage subpopulation, and modeled for each distinct pseudotime branch with spline curves. [file Image6.tif]
