# Supplementary material for: Sex-associated transcriptional changes to synovial macrophages in the aging joint
Source: Front Immunol. 2026 Mar 3;17:1724385. doi: 10.3389/fimmu.2026.1724385 (PMC12992061; doi:10.3389/fimmu.2026.1724385)
Supplement: Supplementary file 7 [file DataSheet1.pdf]

frontiers

The International Society for the Advancement of Cytometry (ISAC) has highlighted the importance of including comprehensive methodological information to ensure data reproducibility and reliability. In line with this, Frontiers in Immunology now requires authors to submit a checklist for manuscripts that involve flow or mass cytometry. This checklist helps standardize the reporting process, improving the quality and transparency of published data. By doing so, we support scientific progress, making it easier for other researchers to replicate and validate experiments.

This form should be submitted with any manuscripts using flow or mass cytometry.

Sample/specimen/material description

- [ ] Total blood
- [ ] PBMCs
- [x] Organ digests

Other____

Did the samples suffer any treatment before or after incubation with the antibodies?

- [ ] Drug _____________________________________
- [ ] Cell permeabilization ______________________
- [x] Dye eBioscience Fixable Viability Dye eFluor 506
- [ ] Propidium iodine
- [ ] Not applicable

Other Purified Rat Anti-Mouse CD16/CD32 (FC Block)

Instrument and antibodies

Name of the Cytometer FACSAriaII

**Antibodies and targets**	**Fluorochrome/ Metal**	**Catalog number/Company**
e.g. anti-CD4	FITC	Cat. XXX/ XXX ltd.
anti-CD64	PE	Cat. 139303/BioLegend

anti-SiglecF	PE-CF594	562757/BD Biosciences
anti-Ly6G	PE-CF594	562700/BD Biosciences
anti-CD4	PE-CF594	562285/BD Biosciences
anti-CD8a	PE-CF594	562283/BD Biosciences
anti-CD19	PE-CF594	562291/BD Biosciences
anti NK1.1	PE-CF594	562864/BD Biosciences
anti MHCII	PE Cy7	107630/BioLegend
anti-CD11b	BB700	566416/BD Biosciences
anti-CD45	FITC	553270/BD Biosciences
anti-CD177	AF647	566599/BD Biosciences
anti-Ly6C	APCCy7	560596/BD Biosciences
anti-CX3CR1	BUV395	567821/BD Biosciences
anti-CD16/CD32	none	553141/BD Biosciences

Data analyses

1. Name of the software FlowJo v10____________________
2. Reference gating strategy in the manuscript or supplementary material

Gating strategy in (eg Figure X) Supplemental Figure 1______
